# A homozygous *FANCM* frameshift pathogenic variant causes male infertility

**DOI:** 10.1038/s41436-018-0015-7

**Published:** 2018-06-12

**Authors:** Hao Yin, Hui Ma, Sajjad Hussain, Huan Zhang, Xuefeng Xie, Long Jiang, Xiaohua Jiang, Furhan Iqbal, Ihtisham Bukhari, Hanwei Jiang, Asim Ali, Liangwen Zhong, Tao Li, Suixing Fan, Beibei Zhang, Jianing Gao, Yang Li, Jabeen Nazish, Teka Khan, Manan Khan, Muhammad Zubair, Qiaomei Hao, Hui Fang, Jun Huang, Mahmoud Huleihel, Jiahao Sha, Tej K. Pandita, Yuanwei Zhang, Qinghua Shi

**Affiliations:** 1 Hefei National Laboratory for Physical Sciences at Microscale, The First Affiliated Hospital of USTC, USTC-SJH Joint Center for Human Reproduction and Genetics, The CAS Key Laboratory of Innate Immunity and Chronic Diseases, School of Life Sciences, CAS Center for Excellence in Molecular Cell Science, Collaborative Innovation Center of Genetics and Development, University of Science and Technology of China, Hefei, 230027 China; 2 Life Sciences Institute, Zhejiang University, Hangzhou, 310058 China; 3 Shraga Segal Department of Microbiology and Immunology, Faculty of Health Sciences, Ben Gurion University of the Negev, Beer Sheva, 84105 Israel; 4 State Key Laboratory of Reproductive Medicine, Nanjing Medical University, Nanjing, 210029 China; 5 Department of Radiation Oncology, The Houston Methodist Research Institute, Houston, TX, 77030 United States

**Keywords:** *FANCM* PV, Male infertility, Spermatogenic failure, Interstrand crosslink sensitivity Fanconi anemia

## Abstract

**Purpose:**

Fanconi anemia (FA) genes play important roles in spermatogenesis. In mice, disruption of *Fancm* impairs male fertility and testicular integrity, but whether *FANCM* pathogenic variants (PV) similarly affect fertility in men is unknown. Here we characterize a Pakistani family having three infertile brothers, two manifesting oligoasthenospermia and one exhibiting azoospermia, born to first-cousin parents. A homozygous PV in *FANCM* (c.1946_1958del, p.P648Lfs*16) was found cosegregating with male infertility. Our objective is to validate that *FANCM* p.P648Lfs*16 is the PV causing infertility in this family.

**Methods:**

Exome and Sanger sequencing were used for PV screening. DNA interstrand crosslink (ICL) sensitivity was assessed in lymphocytes from patients. A mouse model carrying a PV nearly equivalent to that in the patients (*Fancm*^*ΔC/ΔC*^) was generated, followed by functional analysis in spermatogenesis.

**Results:**

The loss-of-function *FANCM* PV increased ICL sensitivity in lymphocytes of patients and *Fancm*^*ΔC/ΔC*^ spermatogonia. Adult *Fancm*^*ΔC/ΔC*^ mice showed spermatogenic failure, with germ cell loss in 50.2% of testicular tubules and round-spermatid maturation arrest in 43.5% of tubules. In addition, neither bone marrow failure nor cancer/tumor was detected in all the patients or adult *Fancm*^*ΔC/ΔC*^ mice.

**Conclusion:**

These findings revealed male infertility to be a novel phenotype of human patients with a biallelic *FANCM* PV.

## Introduction

Male infertility affects approximately 7% of all men^1^ and presents commonly with spermatogenic failure. While genetic factors may be the major underlying cause of spermatogenic failure, in the majority of cases, the etiologies remain undetermined.^2^ Notably, it is estimated that in human ~1,000 genes are expressed in a testis-enriched manner,^3,4^ but only a small number of these genes have been found to contribute to human male infertility so far. Thus, understanding the underlying genetic basis of spermatogenic failure is of great significance for clinical diagnosis and treatment of male infertility.

Fanconi anemia (FA) is a genetic disease with highly variable clinical manifestations, typically described as bone marrow failure, congenital defects, cancer predisposition, and cellular hypersensitivity to DNA interstrand crosslink (ICL)-inducing agents, such as mitomycin C (MMC) (In the OMIM database, the ID for Fanconi Anemia is #227650, and currently 21 genes are related to this disease, which are *FANCA*, *B*, *C*, *D1*, *D2*, *E*, *F*, *G*, *I*, *J*, *L*, *M*, *N*, *O*, *P*, *Q*, *R*, *S*, *T*, *U*, *V, W* (MIM *607139, *300515, *613899, *600185, *613984, *613976, *613897, *602956, *611360, *605882, *608111, *610355, *602774, *133520, *179617, *113705, *610538, *600375, *604094 and *614151, respectively).^5^ Reduced fertility can also be commonly found in FA patients with fertility being impaired in about half of female patients and almost all male patients.^6^ To date, biallelic or X-linked recessive PV in any one of the FA genes (*FANCA*, *B*, *C*, *D1*, *D2*, *E*, *F*, *G*, *I*, *J*, *L*, *M*, *N*, *O*, *P*, *Q*, *R*, *S*, *T*, *U*, *V, W*) ^7–10^ (Fanconi Anemia Mutation Database, http://www.rockefeller.edu/fanconi/), except *FANCM*, have been implicated in the causation of FA. The FA proteins encoded by these genes cooperatively function in a common DNA repair pathway, i.e., the FA pathway, for ICL repair.^11,12^ Upon ICL occurring, FANCM recruits seven additional FA proteins to assemble into the FA core complex, which subsequently monoubiquitinates FANCD2-FANCI to activate downstream FA pathway.^13^ If the function of any FA protein is disrupted, repair of ICLs would be impaired.

FANCM is the only protein within the FA core complex that can recognize and bind to stalled replication forks. The importance of FANCM in mammals was first suggested in 2004, when biallelic *FANCM* PV was identified in a FA patient.^14^ However, it was later found that the patient also harbored biallelic pathogenic *FANCA* PV.^15^ Due to the lack of further reports of biallelic PV in FA patients, it was not clear whether *FANCM* PV are causative for FA until recently when Bogliolo et al..^16^ and Catucci et al.^17^ reported that biallelic *FANCM* PV increased the risks of ICL sensitivity, early-onset cancers, and chemotherapy/radiotherapy-induced pancytopenia, but did not cause bone marrow failure. Similarly, increased tumorigenesis and cellular ICL sensitivity were also detected in *Fancm* knockout (*Fancm*^*∆2/∆2*^) mice, as well as *Fancm*^*C4/C4*^ mice carrying a loss-of-function missense PV in the N-terminal helicase domain,^18,19^ while whether they would develop bone marrow failure was yet undetermined. Moreover, mouse embryonic fibroblasts (MEFs) prepared from these *Fancm* mutant mice had an increased rate of sister chromatid exchange,^18,19^ and non-Mendelian inheritance of *Fancm*^*∆2/∆2*^ alleles in females was observed,^18^ which are atypical phenotypes for FA mice. Hence, *FANCM* appears different from other FA genes and biallelic PV in *FANCM* do not cause FA.

Hypogonadism and reduced fertility have been noted in both male and female mice with homozygous *Fancm* PV,^18,19^ mainly due to defective proliferation of primordial germ cells (PGCs). Similar defects have also been reported in mice carrying other FA mutant genes.^20–22^ In human, 15 individuals—3 men^16^ and 5 women^17^ as well as 7 with unknown gender information^23^—have been reported with biallelic *FANCM* PV. The fertility information for the individuals with unknown gender, and the 3 men and 3 women was not provided. The remaining 2 women had 1 and 2 children, respectively.^17^ Moreover, at least 2 of the female patients were mentioned with early menopause.^17^ Hence, biallelic *FANCM* PV may be associated with impaired fertility in human females, but whether they could cause infertility in men, particularly spermatogenic failure, is yet unknown.

In this study, we identified a homozygous *FANCM* PV (c.1946_1958del, p.P648Lfs*16) in three patients with idiopathic infertility, born to first-cousin parents, from Pakistan. This PV causes lymphocytic ICL sensitivity in patients, thus showing a loss-of-function effect. *Fancm*^*ΔC/ΔC*^ mice, carrying a PV nearly equivalent to that in our patients, also showed spermatogenic failure resulted from a progressive loss of spermatogonial stem cells (SSCs) and partial maturation arrest at the round spermatid stage. Moreover, neither the three patients nor *Fancm*^*ΔC/ΔC*^ mice manifested with bone marrow failure. Altogether, these findings revealed that male infertility is a novel phenotype of the biallelic *FANCM* PV in humans.

## Methods

### Study participants

Three members of a consanguineous Pakistani family were enrolled in this study and written informed consent was received from all participants prior to the onset of the study. Family members IV:I, IV:2, and IV:3 had at least two semen analyses, respectively. Similarly, a single routine blood test was obtained from IV:1, whereas, 2 tests were performed for IV:2 and IV:3 at 2-year intervals in local laboratories.

### Exome sequencing and data analysis

Total genomic DNA (gDNA) was isolated from peripheral blood using the QIAamp DNA Blood Mini Kit (QIAGEN) according to the manufacturer’s instructions. DNA integrity was assessed by 1% agarose gel electrophoresis, and then the DNAs were fragmented using Covaris focused ultrasonication. An Agilent SureSelect Human All Exon v5 Kit was used for the capture of known exons and exon–intron boundary sequences for family members III:1 and IV:1, and sequencing DNA libraries were prepared following the manufacturer’s protocol. Sequencing was performed on a Hiseq2000 platform (Illumina). 3.2 Gb and 3.5 Gb of mappable sequence data were obtained for III:1 and IV:1, respectively, with 99.8% coverage and 54.09× or 65.28× mean depth of target region. Sequencing reads (.qseq format) were aligned to the human genome (GRCh37/hg19) using Burrows–Wheeler Aligner (BWA)^24^ with default parameters. By using SAMtools (http://samtools.sourceforge.net/), the SAM file from each sample was converted to a BAM file, sorted, and merged. Polymerase chain reaction (PCR) duplicates were removed using Picard (http://picard.sourceforge.net/). Files were further processed using a Genome Analysis Toolkit (GATK) from the Broad Institute (http://www.broadinstitute.org/gatk/). All BAM files were locally realigned using indel realigner. Both single-nucleotide variants (SNVs) and indels within the captured coding exonic intervals were called using GATK’s Unified Genotyper.

### Gene filtration

The detected variants were filtered and annotated based on Ensembl (http://www.ensembl.org). Variants within exons or exon–intron boundaries were retained. Variants with genomic frequency MAF >0.05 in the 1000 Genomes Project (ftp://ftp.1000genomes.ebi.ac.uk/vol1/ftp), ExAC (http://exac.broadinstitute.org/) or ESP6500 (http://evs.gs.washington.edu/EVS/) were excluded, followed by elimination of variants that were homozygous in our in-house exome sequencing (ES) data sets from 578 fertile men (41 Pakistanis, 254 Chinese, and 283 Europeans). Functional annotation for genes harboring variants was performed based on our database, SpermatogenesisOnline 1.0,^25^ and published literature; variants in genes that have no function in spermatogenesis according to SpermatogenesisOnline 1.0 (probability ≤0.3) and literature were removed. Variants in genes that are expressed in testis ≥1 FPKM (and in at least one tissue >10 FPKM) at messenger RNA (mRNA) level^4^ or can be detected in testis at protein level^3^ were kept. Variants predicted to be nondeleterious by >50% software (Supplementary Table S1) covering them were omitted. The variants left out (Supplementary Table S2) were detected by Sanger sequencing in all of the available family members. The ES data has been deposited in ArrayExpress under accession number E-MTAB-6090. Supplementary Figure S1 shows a flow chart of the gene filtration process.

### Chromosomal breakage assay in cultured human peripheral blood lymphocytes

Peripheral blood lymphocyte cultures were established as we reported^26^ and incubated with different concentrations of MMC (0 nM, 50 nM, 150 nM, 300 nM) for 72 h. For each concentration of MMC, two cultures were set up per donor. Metaphase spreads were prepared using standard cytogenetic techniques. The sample size was determined by power analysis. Two independent experiments were performed with at least 40 cells scored per group.

### Western blot

Whole blood lysates were prepared from blood samples stored in TRIzol^®^ reagent (Thermo Fisher Scientific). Briefly, chloroform was added to partition proteins into the organic phase. After removing the aqueous phase, the organic phase was mixed with ethanol and centrifuged at 12,000 rpm for 10 min at 4 °C. The upper phase was transferred into a new tube and proteins were precipitated with isopropanol. The protein pellet was rinsed with ethanol (with 0.3 M guanidine hydrochloride) and dissolved in 1% SDS. Antibodies used are listed in Supplementary Table S3.

To obtain cell lysates, HEK293T cells were transfected with EGFP-FANCM-WT or EGFP-FANCM-MUT, respectively. Twenty-four hours later, the cells were lyzed with SDS lysis buffer and boiled for 10 min. The proteins were then separated on a 10% SDS polyacrylamide gel by electrophoresis for Western blotting as we described.^27^ Vendors and catalog numbers of the antibodies used are listed in Supplementary Table S3.

### Animal studies

All the animal studies were performed in C57BL/6 mice (*Mus musculus*) and details for the experiments are described in Supplementary Methods.

### Statistics

All data were analyzed using GraphPad Prism 5 (GraphPad Software). Statistical analyses were performed by two-tailed Student’s *t*-test for comparison between two groups and by analysis of variance (ANOVA) test for comparison among multiple groups to determine the significances. *P* values of less than 0.05 were considered statistically significant.

### Study approval

All human studies have been approved by the institutional human ethics committee at the University of Science and Technology of China (USTC) with the approval number USTCEC20140003. All experiments involving animals were approved by the institutional animal ethics committee at USTC with the approval number USTCACUC1301021.

## Results

### Clinical phenotypes

**Fig. 1:**
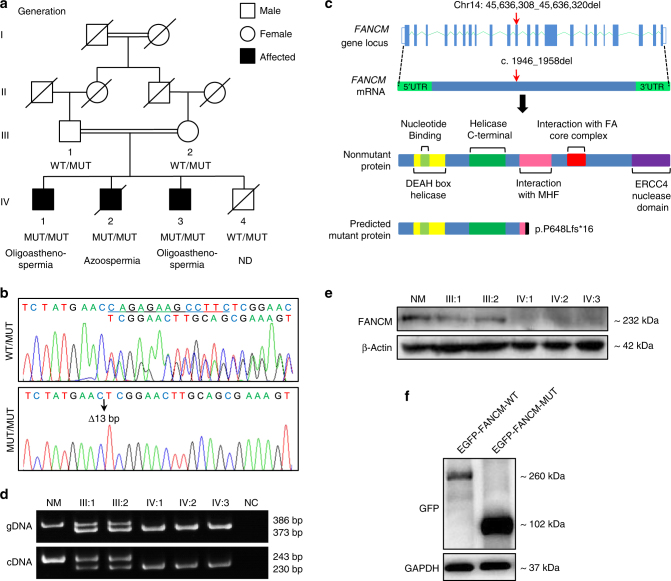
Identification of a *FANCM* frameshift PV in a consanguineous family with male infertility. **a** Segregation of the *FANCM* frameshift PV in a Pakistani consanguineous family. Slashes denote deceased family members and double horizontal lines represent consanguineous marriages. *WT* wildtype, *MUT* mutant, *ND* not determined. **b** Representative chromatograms of the *FANCM* frameshift PV (c.1948_1956del) in patients (MUT/MUT) and carriers (WT/MUT). **c** The *FANCM* PV was supposed to cause a frameshift at codon 648 and introduce a premature stop codon at codon 664, resulting in a predicted truncated protein of 663 aa (p.P648Lfs*16). **d** Polymerase chain reaction (PCR) assays confirmed the 13-nucleotide deletion PV of *FANCM* in genomic DNA (gDNA) and complementary DNA (cDNA) from blood samples of patients and their parents. A fertile individual served as the normal control (NM) and ddH_2_O as the negative control (NC). **e** Western blot analysis failed to detect the presence of FANCM protein in blood samples from the infertile brothers, using an anti-FANCM antibody recognizing an epitope located between amino acid 600 and 700. β-Actin served as a loading control. **f** EGFP-FANCM-MUT produced a ~102 kDa fusion protein corresponding to the size of predicted truncated FANCM (~75 kDa) fused to EGFP (~27 kDa)

We investigated the genetic cause of male infertility in a Pakistani family with three infertile brothers, who were born to a first-cousin marriage (Fig. 1a). All three patients have a normal 46,XY karyotype and none of them have any history of tumor/cancer, drinking, or smoking. Two of the affected brothers, IV:1 (married for 21 years) and IV:3 (single) were diagnosed with mild and severe oligoasthenospermia, respectively. Both had largely normal plasma levels of testosterone and pituitary hormones. The third affected brother IV:2 (married for 9 years) was azoospermic and was diagnosed with primary valvular heart disease in his teens. He had two open-heart surgeries for artificial valve replacements, one at 17 years old and the second at 37 years old, and he died of an acute heart attack caused by blood clots in the heart valves at 42 years old (during the preparation of manuscript). The youngest brother IV:4 (single) died from primary valvular heart disease at 34 years of age before semen analysis could be performed. The clinical findings of the affected brothers are summarized in Table 1.

**Table 1:** Clinical characteristics of patients

	*Reference values*	*IV:1*	*IV:2*	*IV:3*	*IV:4*	*III:1*
Age (years)^a^		48	42	37	34	72
Height/weight (cm/kg)		165/65	165/55	169/46	—	164/65
Karyotype		46,XY	46,XY	46,XY	—	46,XY
Heart disease		No	Valvular heart disease	No	Valvular heart disease	ND
Semen analysis^b—^						
Semen volume (ml)	>1.5 ml	1.4 ± 0.6	1.6 ± 1.0	2.4 ± 0.2	—	—
Sperm count (millions/ml)	>15	10.3 ± 2.0	0	0.1 ± 0.1	—	—
Motile sperm (%)	>40	20.0 ± 11.6	0	31.3 ± 31.3	—	—
Progressively motile sperm (%)	>32	3.3 ± 3.3	0	-		
Morphologically normal sperm (%)	>4	74.7 ± 3.3	0	32.5 ± 32.5	—	—
Hormone analysis^c^						
Testosterone (ng/dl)	249-836	299.4	218.0	383.4	—	ND
FSH (mIU/ml)	1.4–15.4	12.2	19.7	16.3	—	ND
LH (mIU/ml)	1.2–7.8	5.1	ND	6.3	—	ND
Prolactin (ng/ml)	3–14.7	11.1	ND	5.2	—	ND
TSH (μIU/ml)	0.4–4.2	2.1	ND	1.1	—	ND
Routine blood test^c,d^						
Hemoglobin (g/dl)	13.7–16.3	14.8	13.9 ± 0.4	15.9 ± 0.8	—	12.3 ± 0.1
RBC count (×10^12^/L)	4.5–6.5	5.9	4.68 ± 0.3	5.4 ± 0.2	—	5.0 ± 0.0
HCT (%)	41.9–48.7	48.5	42.1 ± 2.5	47.5 ± 0.4	—	38.8 ± 0.3
MCV (fl)	76–96	81.6	89.6 ± 1.5	86.8 ± 2.3	—	77.8 ± 0.2
MCH (pg)	26–32	25.1	29.0 ± 2.0	29.0 ± 0.3	—	24.5 ± 0.7
MCHC (g/dl)	32–36	30.7	32.9 ± 1.3	33.4 ± 1.4	—	31.5 ± 0.8
WBC (×10^9^/L)	4–10	9.7	5.0 ± 0.1	9.0 ± 1.4	—	11.5 ± 1.0
Neutrophils (%)	40–75	69.0	59.7 ± 2.3	48.5 ± 1.5	—	64.8 ± 12.2
Lymphocytes (%)	20–45	25.0	23.5 ± 0.5	36.5 ± 7.5	—	22.9 ± 6.9
Eosinophils (%)	1–6	4.0	7.7 ± 3.4	10.0 ± 5.00	—	5.6 ± 2.6
Monocytes (%)	2–10	2.0	9.1 ± 5.1	5.0 ± 1.0	—	6.4 ± 2.4
Platelets (×10^9^/L)	150–400	233.0	248.0 ± 12.00	278.0 ± 8.0	—	369.0 ± 28.0

*ND* not determined, *RBC* red blood cell, *HCT* hematocrit, *MCV* mean corpuscular volume, *MCH* mean corpuscular hemoglobin, *MCHC* mean corpuscular hemoglobin concentration, *WBC* white blood cell count, *FSH* follicle-stimulating hormone, *LH* luteinizing hormone, *TSH* thyroid-stimulating hormone

^a^Ages of death for IV:2 and IV:4 and ages at the manuscript submission for others

^b^Reference values were published by the World Health Organization (WHO) in 2010

^c^Reference values were suggested by local clinical laboratory

^d^Blood tests were done twice, 2 years apart by two different clinics, except for IV:1, who refused to take a second test. IV:4 was deceased before tests could be performed. Data are expressed as mean ± SEM

### Identification of a homozygous *FANCM* PV in infertile patients

To identify the genetic cause of male infertility in this family, we performed exome sequencing (ES) in patient IV:1 and his father (III:1). Supplementary Figure S1 describes the analysis of ES data. Briefly, variants with genomic frequency MAF >0.05 in human genetic variation databases (1000 Genomes, ESP6500, or ExAC) or homozygous in our 578 in-house fertile male controls were excluded, resulting in 121 variants in 111 genes. After removal of variants in genes that have no functional roles in spermatogenesis based on SpermatogenesisOnline 1.0^25^ and literature, variants in genes that are detected at the mRNA level (≥1 FPKM in testes and >10 FPKM in at least one tissue)^4^ or at the protein level in testes^3^ were kept. Variants predicted to be nondeleterious were excluded, reducing the number to 7 variants in 7 genes. Sanger sequencing for these 7 variants was performed in all the available family members and identified 2 variants, *FANCM* c.1946_1958del and X-linked *TAF7L* c.1047_1052delGGATGA, recessively cosegregating with male infertility in the family (Supplementary Table S2, Fig. 1b, and Supplementary Figure S2). Further manual literature search revealed that *TAF7L* c.1047_1052delGGATGA was present in fertile men at a frequency of 25%.^28^ Thus, we considered that the PV in *FANCM* was the only candidate PV causing male infertility in this family.

The PV is a 13-nucleotide deletion in exon 11 of *FANCM* and would cause a frameshift at codon 648 that introduces a premature stop codon at codon 664, resulting in a predicted truncated protein of 663 aa (p.P648Lfs*16) (Fig. 1c). This PV was confirmed by PCR at both the gDNA and complementary DNA (cDNA) levels to be homozygous in all three infertile patients and heterozygous in their parents (Fig. 1d). Western blot analysis using an antibody recognizing an epitope between FANCM amino acids 600 and 700 confirmed the absence of full-length FANCM protein from the blood samples of all three infertile brothers (Fig. 1e). Recombinant expression of the mutant FANCM (FANCM-MUT) coding DNA sequence (CDS) cloned downstream of an enhanced green fluoresent protein (EGFP) reporter CDS yielded GFP signals (Supplementary Figure S3a) and a predicted truncated fusion protein of 102 kDa (75 kDa for FANCM-MUT plus 27 kDa for EGFP) (Fig. 1f). However, insertion of FANCM-MUT CDS upstream of EGFP did not produce GFP signals (Supplementary Figure S3b). Taken together, these results suggested that the PV in patients could result in a truncated FANCM protein.

### Increased lymphocytic ICL sensitivity in patients

**Fig. 2:**
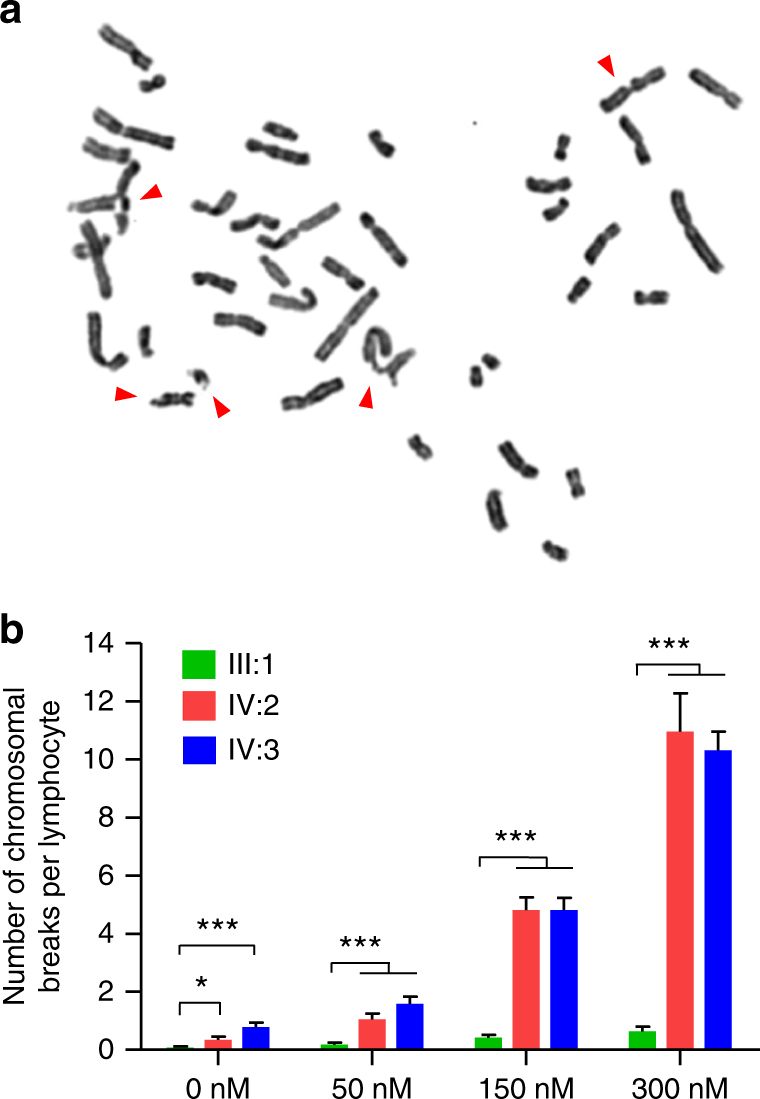
*FANCM* PV impairs DNA break repair in patients. **a** A representative cultured metaphase lymphocyte from patients after 300 nM MMC treatment. Chromosomal breaks are indicated by arrowheads. **b** Quantification of chromosomal breaks in lymphocytes from patients (VI:2 and VI:3) and their father (III:1) after MMC treatment range from 0 nM to 300 nM. At least 40 cells were scored for each group. Data are represented as mean ± SEM from at least two independent experiments. *p *<* 0.05, ***p *<* 0.001; one-way analysis of variance (ANOVA) test

Disruption of FANCM causes deficient repair of DNA damages induced by ICL agents, which can manifest as an increase in chromosomal breaks.^15,18^ To determine whether our PV disrupts the function of FANCM, we evaluated DNA break repair in cultured peripheral blood lymphocytes from patients IV:2 and IV:3 (IV:1 refused to take the test) and their father (Fig. 2). Lymphocytes from the father displayed few chromosomal breaks after treatment with 50 nM, 150 nM, or 300 nM MMC; however, lymphocytes from both IV:2 and IV:3 displayed a dose-dependent increase in MMC-induced chromosomal breaks per cell, which averaged 3 to 17 times those of the father (Fig. 2), functionally indicating the existence of the PV, and its disruption of FANCM function in the patients.

### The *FANCM* PV impairs FA pathway

The truncated FANCM (p.P648Lfs*16) is predicted to lose the domains required for its interaction with the FA core complex (Fig. 1c),^14,29,30^ and thus it should not activate FANCD2 monoubiquitination (FANCD2-L). To test this, we first generated *FANCM*^*-/-*^ HEK293T cells (Supplementary Figure S4a,b), and then expressed the wild type and mutant FANCM cDNAs in these cells, respectively, followed by the detection of FANCD2 and its monoubiquitination, as well as γH2AX, a marker for DNA breaks. As expected, and in comparison to *FANCM*^*-/-*^ cells expressing FANCM-WT, FANCD2 focus formation was impaired and the level of FANCD2-L decreased in *FANCM*^*-/-*^ cells expressing FANCM-MUT after MMC treatment, which was very similar to those observed in *FANCM* null cells (Supplementary Figure S4c,d). γH2AX foci (Supplementary Figure S4e) and protein (Figure S4f) dramatically increased in *FANCM*^*-/-*^ cells expressing FANCM-MUT. Furthermore, similar to lymphocytes of the patients (Fig. 2), *FANCM*^*-/-*^ cells expressing FANCM-MUT showed significantly increased chromosomal breaks after MMC treatment (Supplementary Figure S4g). These results indicated that the truncating *FANCM* PV impairs FA pathway, demonstrating a loss-of-function effect.

### Spermatogenic failure in *Fancm*^*ΔC/ΔC*^ mice

**Fig. 3:**
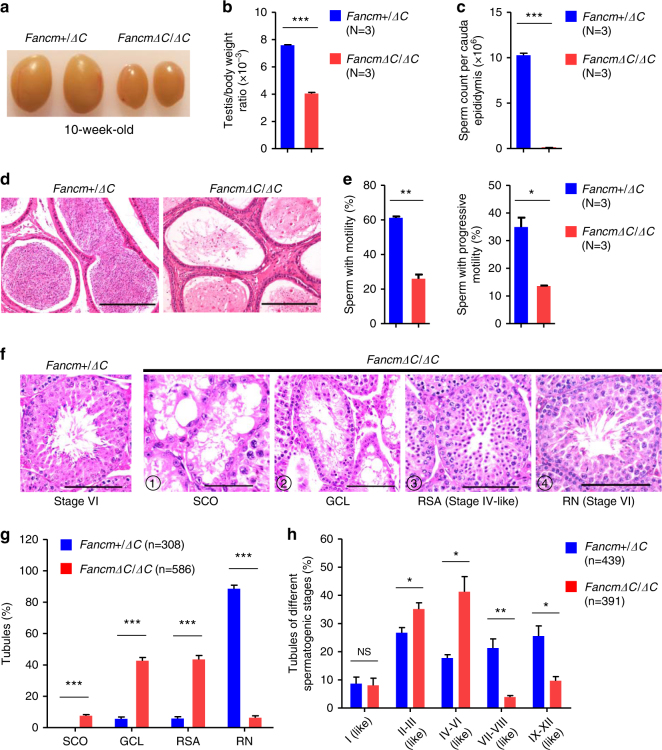
Spermatogenic failure in *Fancm*^*ΔC/ΔC*^ mice. **a** Representative images of testes from 10-week-old *Fancm*^*+/ΔC*^ and *Fancm*^*ΔC/ΔC*^ mice. **b** Testis/body weight ratio; (**c**,**d**) sperm number, motile sperm; and **e** progressively motile sperm in cauda epididymis of adult *Fancm*^*ΔC/ΔC*^ mice significantly decreased. **f** Representative images of H&E stained testicular sections showing various seminiferous tubules in adult *Fancm*^*ΔC/ΔC*^ mice. ① Sertoli cell–only tubules (SCO), ② degenerated tubules with massive germ cell loss (GCL), ③ tubules with round spermatids as their most advanced spermatogenic cells (round spermatid arrest, RSA), ④ relatively normal tubules (RN). Scale bar = 100 μm. **g** Quantification of different types of seminiferous tubules in *Fancm*^*+/ΔC*^ mice and *Fancm*^*ΔC/ΔC*^ mice. **h** Quantification of seminiferous tubules of different spermatogenic stages in adult mice. Spermatogenic stages were divided into five groups based on the presence and arrangement of spermatogenic cells in anti-PNA-stained testicular sections: I, II–III, IV–VI, VII–VIII, and IX–XII. Data are represented as mean ± SEM from at least three independent experiments. *N* the number of mice examined, *n* the number of tubules examined. * *p* *<* 0.05, **p < 0.01, ***p < 0.001; Student’s *t*-test. NS no significance

Because the patients declined to provide testicular tissues, and to understand whether the *FANCM* p.P648Lfs*16 PV is indeed the cause of male infertility, we generated a mouse model with a homozygous *Fancm* p.R638Rfs*8 PV (*Fancm*^*ΔC/ΔC*^) nearly equivalent to that in our patients (Supplementary Figure S5). In fertility tests, adult *Fancm*^*ΔC/ΔC*^ males were generally subfertile (whereas one mouse was infertile) and produced significantly smaller litters than WT and *Fancm*^*+/ΔC*^ mice (Supplementary Table S4). They had an approximately 47% reduction in testis size (Fig. 3a, b) and a 97-fold reduction in cauda epididymal sperm count when compared with their heterozygous littermates (Fig. 3c, d). We next examined sperm motility using a computer-assisted sperm analyzer, and found a significant decrease in the proportion of motile and progressively motile sperm in *Fancm*^*ΔC/ΔC*^ mice (Fig. 4e). Moreover, *Fancm*^*ΔC/ΔC*^ mice exhibited a 26-fold increase in the proportion of morphologically abnormal sperm (Supplementary Figure S6). Hence, *Fancm*^*ΔC/ΔC*^ mice phenocopied the clinical phenotypes in our patients (Table 1).

### Degenerated testicular tubules in *Fancm*^*ΔC/ΔC*^ mice

To further investigate the effects of the mutant FANCM on spermatogenesis, we compared the testicular histology between *Fancm*^*+/ΔC*^ and *Fancm*^*ΔC/ΔC*^ mice. Testicular tubules from adult *Fancm*^*+/ΔC*^ mice had normal architecture with well-organized distribution of germ cells (Fig. 3f). In contrast, testicular tubules from adult *Fancm*^*ΔC/ΔC*^ mice were predominantly degenerated (Fig. 3f) accompanied with increased apoptosis of germ cells (Supplementary Figure S7). Additionally, 7.6% of *Fancm*^*ΔC/ΔC*^ testicular tubules were Sertoli cell only (SCO), and 42.6% of tubules exhibited massive loss of germ cells of multiple stages (Fig. 3g), which could result from depletion of SSCs. To ascertain whether SSCs were reduced in *Fancm*^*ΔC/ΔC*^ mice, we performed immunostaining of testicular sections for GFRA1, a common marker for SSCs,^31^ and SOX9, a Sertoli cell marker. The average ratio of GFRA1^+^ cell number to SOX9^+^ cell number per tubule in *Fancm*^*ΔC/ΔC*^ testes was only half of that in control mice at 10 weeks old (Supplementary Figure S8a,b) and was less than one-fourth of that in the control at 70 weeks old (Supplementary Figure S8c), suggesting an age-dependent progressive loss of SSCs in *Fancm*^*ΔC/ΔC*^ mice. Furthermore, spermatogonia of *Fancm*^*ΔC/ΔC*^ males displayed elevated MMC sensitivity (Supplementary Figure S8d), similar to lymphocytes of patients (Fig. 2) and HEK293T cells expressing FANCM-MUT (Supplementary Figure S4g).

Moreover, 43.5% of *Fancm*^*ΔC/ΔC*^ testicular tubules had round spermatids as their most advanced spermatogenic cells (Fig. 3g). Spermatogenic stages of the tubules were further determined based on the pattern of peanut agglutinin (PNA) staining, a marker for acrosome of spermatids (Supplementary Figure S9a). In contrast to *Fancm*^*+/Δ*^ testes, adult *Fancm*^*Δ/Δ*^ testes contained a large proportion of atypical stage II–VI tubules that lacked elongating/ed spermatids, which should present in normal stage II–VI seminiferous tubules (Supplementary Figure S9b). Noticeably, the frequencies of these stage II–VI-like tubules in *Fancm*^*ΔC/ΔC*^ testes were significantly increased, while the frequencies of stage VII–XII tubules were significantly reduced, when compared with those of stage II–VI and VII–XII tubules in control mice respectively (Fig. 3h), further indicating that the homozygous *Fancm* PV caused a partial maturation arrest at the round spermatid stage. Altogether, these observations demonstrated that the homozygous *Fancm* PV disrupted testicular integrity.

### Patients and adult *Fancm*^*ΔC/ΔC*^ mice do not have bone marrow failure or cancer/tumor

Because patients with biallelic *FANCM* PV were recently reported with early-onset cancers but without bone marrow failure,^16,17^ the typical malignancy of FA, we thus investigated whether our *FANCM* PV could cause bone marrow failure in our patients and mice. Routine blood tests were performed in patients and showed that blood cell counts and hemoglobin levels in patients IV:2 and IV:3 were within normal ranges; for patient IV:1, only one blood test was performed and all the values were in the normal range except the mean corpuscular hemoglobin (MCH) and mean corpuscular hemoglobin concentration (MCHC) levels, which were slightly below the normal range (Table 1). Similarly, adult (10-week-old) *Fancm*^*ΔC/ΔC*^ mice did not display significant differences in all the parameters examined including counts of blood cells and hemoglobin levels from those in *Fancm*^*+/+*^ and *Fancm*^*+/ΔC*^ males either (Supplementary Table S5). These findings showed that this *FANCM* PV did not cause bone marrow failure in patients or mice. Interestingly, neither our patients (until the fourth or fifth decade of life) nor *Fancm*^*ΔC/ΔC*^ mice (up to 1 year old) had been detected with any type of cancers/tumors. Hence, although the observation of cellular ICL sensitivity and male infertility in patients might be suggestive of FA, it appears that the homozygous *FANCM* PV do not cause FA.

## Discussion

In the current study, we identified an autosomal recessive *FANCM* p.P648Lfs*16 PV in a first-cousin marriage family, which was homozygous in three infertile brothers with spermatogenic failure and heterozygous in their parents. Patients exhibited increased ICL sensitivity, but not bone marrow failrue or cancer/tumor untill the fourth or fifth decade of life. Mice carrying a PV nearly equivalent to that in our patients showed similar phenotypes, demonstrating that the *FANCM* p.P648Lfs*16 is a loss-of-function PV and causes male infertility recessively. Biallelic *FANCM* PV have been reported in three other human males, but their fertility status is unknown.^16^ Thus, this is the first time a homozygous loss-of-function *FANCM* PV has been reported causing human male infertility by impairing spermatogenesis.

Similar to previous *Fancm* mutant mice,^18,19^ *Fancm*^*ΔC/ΔC*^ testes displayed SCO tubules and a progressive loss of germ cells, a common phenotype shared by all FA mouse models,^20–22^ which is supposed to result from defective repair of ICLs occurring in DNA replication of germ cells. Our finding that proliferating spermatogonia of *Fancm*^*ΔC/ΔC*^ mice showed MMC hypersensitivity first substantiated this supposition. Notably, 43.5% of seminiferous tubules displayed maturation arrest at round spermatid stage in *Fancm*^*ΔC/ΔC*^ mice, which has not been mentioned in *Fancm* mutant mice^18,19^ or reported in any other FA mouse models,^19–22^ suggesting that this role of FANCM could be independent of the canonical FA pathway. Considering that FANCM is required for DNA break repair in mitotic cells and repair of DNA breaks is essential for chromatin remodelling during spermiogenesis,^32^ it would be interesting to know whether FANCM is required for DNA break repair in spermatids, which will be a catalyst for multiple avenues of further research in this poorly understood field.

Congenital defects were not observed in all our patients, except IV:2, who died of valvular heart disease. However, the youngest brother (IV:4) in this family also died of valvular heart disease and he was heterozygous for the *FANCM* PV. Given the consanguinity of this family, it can be inferred that some other PV, at least not the homozygous *FANCM* PV alone, contributed to the valvular heart disease in IV:2. Moreover, consistent with previous reports,^16,17^ our patients show increased ICL sensitivity but do not have bone marrow failure. Similar results were also found in *Fancm*^*ΔC/ΔC*^ mice. All the eight patients previously reported with biallelic *FANCM* PV suffered from cancers;^16,17^ however, none of our three patients has developed any cancer up to the fourth or fifth decade of their life. Moreover, our *Fancm*^*ΔC/ΔC*^ mice have not developed tumors even though they are 1 year old. However, it is not yet clear whether patients IV:1 and IV:3, as well as our homozygous mutant mice, will develop tumors/cancers during their remaining lifespan. This phenotypic discrepancy we observe may result from the difference of PV, where our patients harbor a homozygous p.P648Lfs*16 PV while the reported patients had a homozygous p.Lys863Ilefs*12, p.Ile503*, p.Arg658*, p.Gln1701*, or p.Arg1931* PV^16,17^. These results also signify that biallelic truncating *FANCM* PV could lead to a variety of phenotypes.

It has been reported that patients with ICL-induced chromosomal instability exhibited infertility,^33,34^ and lymphocytes of infertile men showed an increased risk of MMC sensitivity,^35^ implying that defective ICL repair and male infertility are correlated. Our patients exhibited both increased lymphocytic ICL sensitivity and spermatogenic failure, further indicating the importance of ICL repair in both lymphocytes and germ cells. Hence, it would be of great significance to determine the PV frequency of ICL repair genes, such as FA genes, in patients with spermatogenic failure.

In summary, the clinical phenotype spectrum of the patients with a biallelic truncating *FANCM* PV has not been fully defined. Therefore, our findings that a homozygous loss-of-function *FANCM* PV impaired spermatogenesis and caused male infertility provide novel insights into genotype–phenotype correlations for biallelic *FANCM* PV. These findings will improve clinicians’ ability to make an early and accurate diagnosis and facilitate genetic counseling, which will directly benefit the families with affected individuals.

## Electronic supplementary material

Supplementary Materials
