# Correction: A homozygous *FANCM* frameshift pathogenic variant causes male infertility

**DOI:** 10.1038/s41436-018-0127-0

**Published:** 2018-08-29

**Authors:** Hao Yin, Hui Ma, Sajjad Hussain, Huan Zhang, Xuefeng Xie, Long Jiang, Xiaohua Jiang, Furhan Iqbal, Ihtisham Bukhari, Hanwei Jiang, Asim Ali, Liangwen Zhong, Tao Li, Suixing Fan, Beibei Zhang, Jianing Gao, Yang Li, Jabeen Nazish, Teka Khan, Manan Khan, Muhammad Zubair, Qiaomei Hao, Hui Fang, Jun Huang, Mahmoud Huleihel, Jiahao Sha, Tej K. Pandita, Yuanwei Zhang, Qinghua Shi

**Affiliations:** 1 Hefei National Laboratory for Physical Sciences at Microscale, The First Affiliated Hospital of USTC, USTC-SJH Joint Center for Human Reproduction and Genetics, The CAS Key Laboratory of Innate Immunity and Chronic Diseases, School of Life Sciences, CAS Center for Excellence in Molecular Cell Science, Collaborative Innovation Center of Genetics and Development, University of Science and Technology of China, Hefei 230027, China; 2 Life Sciences Institute, Zhejiang University, Hangzhou 310058, China; 3 Shraga Segal Department of Microbiology and Immunology, Faculty of Health Sciences, Ben Gurion University of the Negev, Beer Sheva 84105, Israel; 4 State Key Laboratory of Reproductive Medicine, Nanjing Medical University, Nanjing 210029, China; 5 Department of Radiation Oncology, The Houston Methodist Research Institute, Houston, Texas 77030 USA

**Correction to**: *Genetics in Medicine*, 10.1038/s41436-018-0015-7, published online 12 June 2018

Hao Win, Hui Ma and Sajjad Hussain were incorrectly affiliated to ‘Department of Radiation Oncology, The Houston Methodist Research Institute, Houston, TX 77030 USA’. These authors should only have been affiliated to ‘Hefei National Laboratory for Physical Sciences at Microscale, The First Affiliated Hospital of USTC, USTC-SJH Joint Center for Human Reproduction and Genetics, The CAS Key Laboratory of Innate Immunity and Chronic Diseases, School of Life Sciences, CAS Center for Excellence in Molecular Cell Science, Collaborative Innovation Center of Genetics and Development, University of Science and Technology of China, Hefei 230027, China’. They were also not noted as contributing equally to the paper. Both these errors have now been corrected in the PDF and HTML versions of the paper.

