# Supplementary material for: A homozygous FANCM frameshift pathogenic variant causes male infertility
Source: Genet Med. 2018 Jun 12;21(1):62–70. doi: 10.1038/s41436-018-0015-7 (PMC6752308; doi:10.1038/s41436-018-0015-7)
Supplement: Supplementary file 1 — Supplementary Materials [file 41436_2018_15_MOESM1_ESM.pdf]

Supplementary Materials for

A homozygous *FANCM* frameshift mutation causes male infertility

This PDF file includes:

1. **Supplementary Figure S1-S9**
2. **Supplementary Table S1-S6**
3. **Supplementary Methods**
4. **References**

1. Supplementary Figures

Supplementary Figure S1

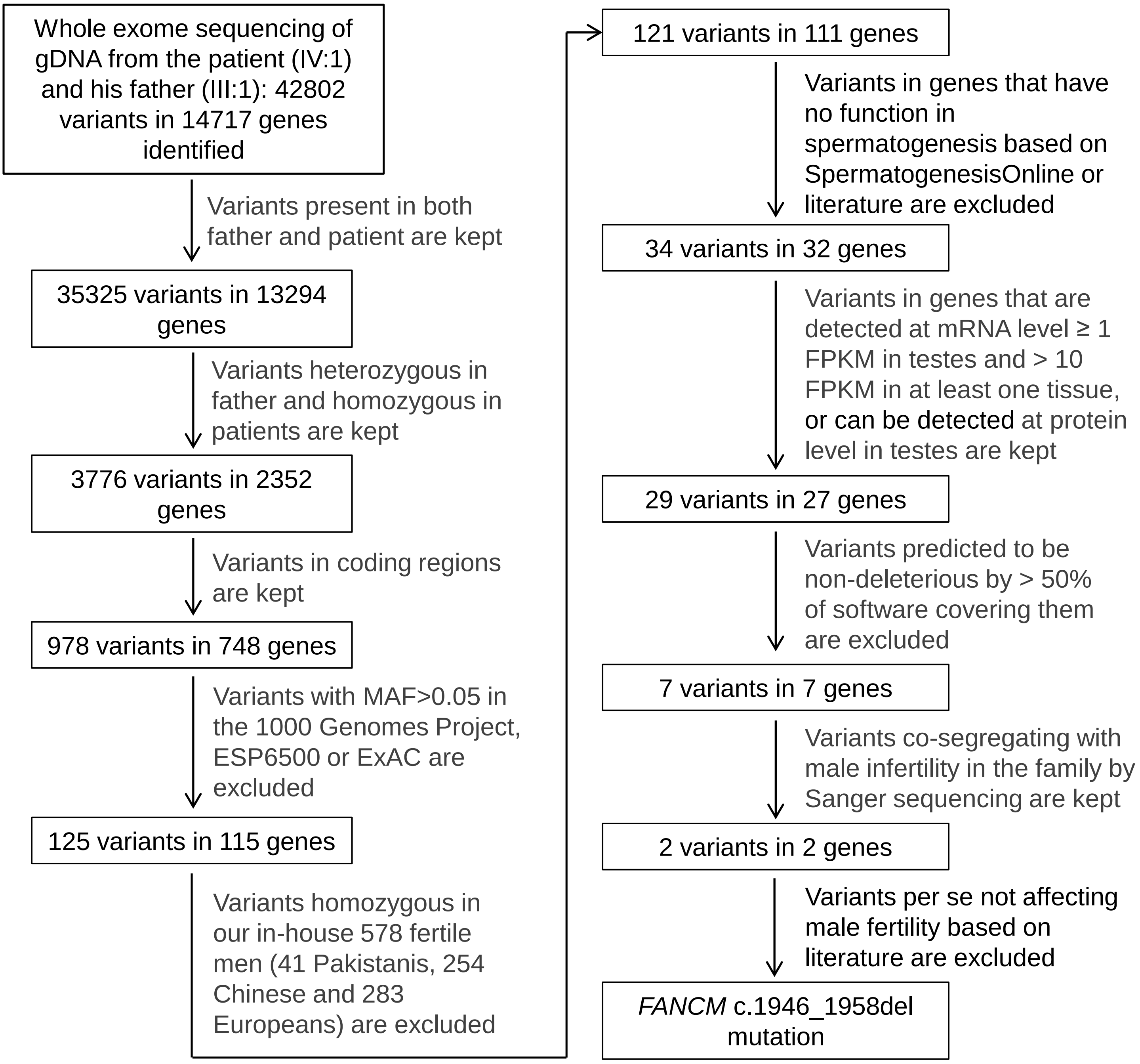

Supplementary Figure S1. Flow chart of whole-exome sequencing data analysis and Sanger sequencing for candidate disease-causing mutations.

Supplementary Figure S2

Family member	Diagnosis	Sanger Sequencing<br>WT *: ATGAACCAGAGAAGCCTTCTCGG<br>MUT: ATGAACTCGGAACTTGCAGCGAA	Genotype
III:1	-	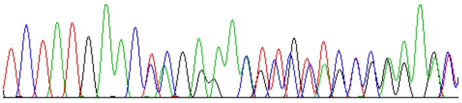	WT/MUT
III:2	-	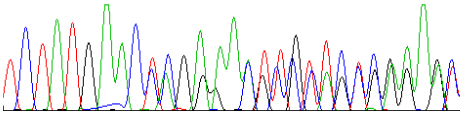	WT/MUT
IV:1	Oligoastheno-spermia	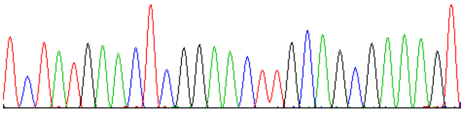	MUT/MUT
IV:2	Azoospermia	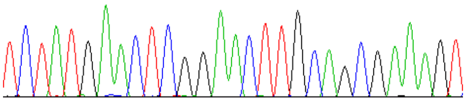	MUT/MUT
IV:3	Oligoastheno-spermia	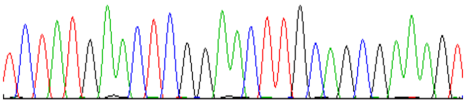	MUT/MUT
IV:4	Undetermined	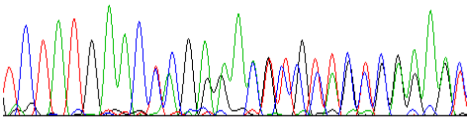	WT/MUT

Supplementary Figure S2. Sequence chromatograms of the *FANCM* frameshift mutation in family members. Deleted nucleotides are marked in red in the WT(*) sequence. WT, wild-type; MUT, mutant.

Supplementary Figure S3

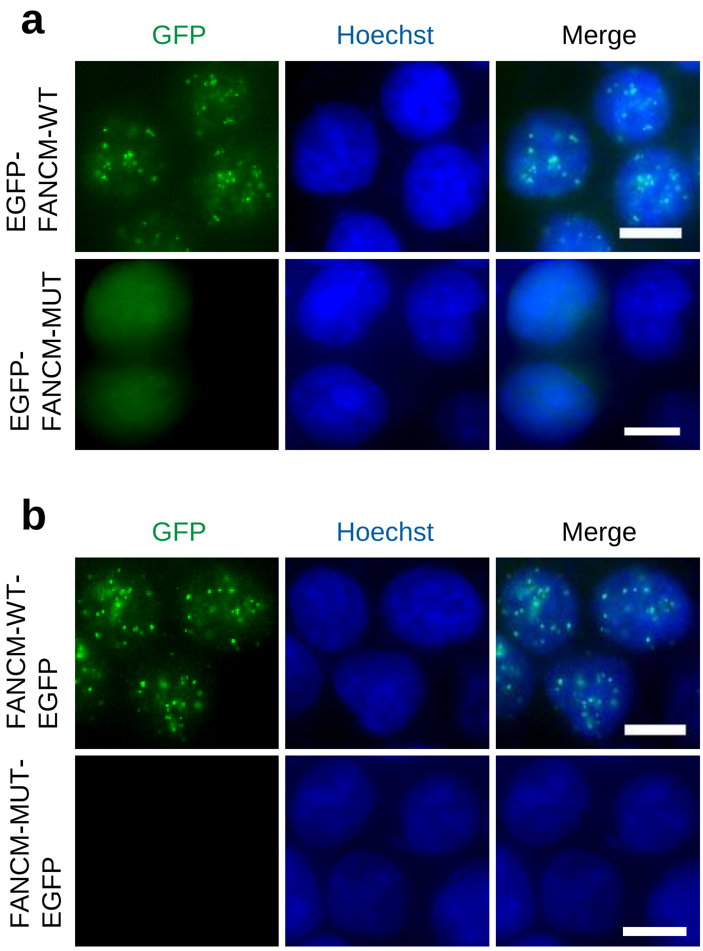

Supplementary Figure S3. *FANCM* c.1946_1958del results in a premature stop codon. (**a**) Representative images showing that exogenous EGFP-FANCM-MUT expressed GFP but could not form nuclear foci in response to MMC treatment (50 ng/ml) in HEK293T cells. Scale bar, 10 μm. (**b**) Representative images showing that GFP fluorescence can be observed in HEK293T cells transfected with FANCM-WT-EGFP, but not in those transfected with FANCM-MUT-EGFP.

Supplementary Figure S4

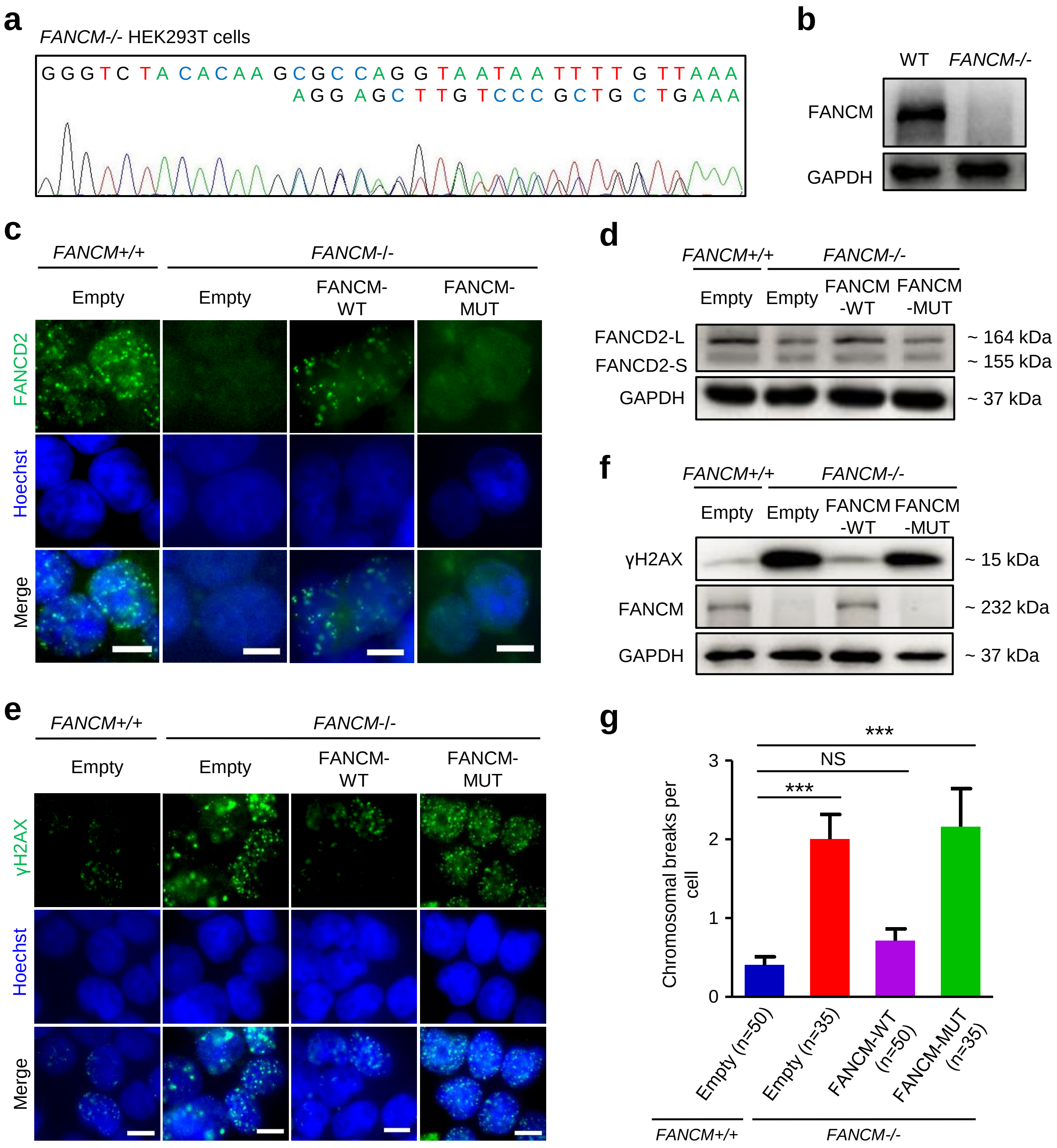

Supplementary Figure S4. Mutant FANCM compromises MMC-induced chromosomal damage repair in HEK293T cells. (**a**) Sanger sequencing of genomic DNA from the selected *FANCM*$^{-/-}$ clone, in which one allele contained a 155 nt deletion (c.522_676del) and the other allele contained both a 79 nt deletion and a one-nucleotide insertion mutation (c. [521_599del: c.677insT]), resulting in predicted truncated proteins p. [Ala174Alafs*15] and p. [Ala174Glufs*9] respectively. (**b**) Western blot analysis failed to detect the presence of FANCM protein using the antibody that recognizes amino acids 600-700 in FANCM (NB110-98674, Novus Biologicals) in *FANCM*$^{-/-}$ cells. GAPDH served as a loading control. (**c** - **f**) In *FANCM*$^{-/-}$ HEK293T cells transfected with FANCM-MUT, FANCD2 foci formation was inhibited (**c**), mono-ubiquitinated FANCD2 (FANCD2-L) decreased (**d**), γH2AX foci (**e**) and γH2AX protein level (**f**) increased after MMC treatment. Scale bar, 10 μm. (**g**) The deficiency in repair of chromosomal breaks in *FANCM*$^{-/-}$ cells induced by MMC (20 ng/ml, 24 hrs) was restored following transfection of FANCM-WT but not FANCM-MUT. Empty, empty vectors. Data are represented as mean ± SEM from at least three experiments; n, the number of cells examined. ***$p < 0.001$; one-way ANOVA. NS, no significance.

Supplementary Figure S5

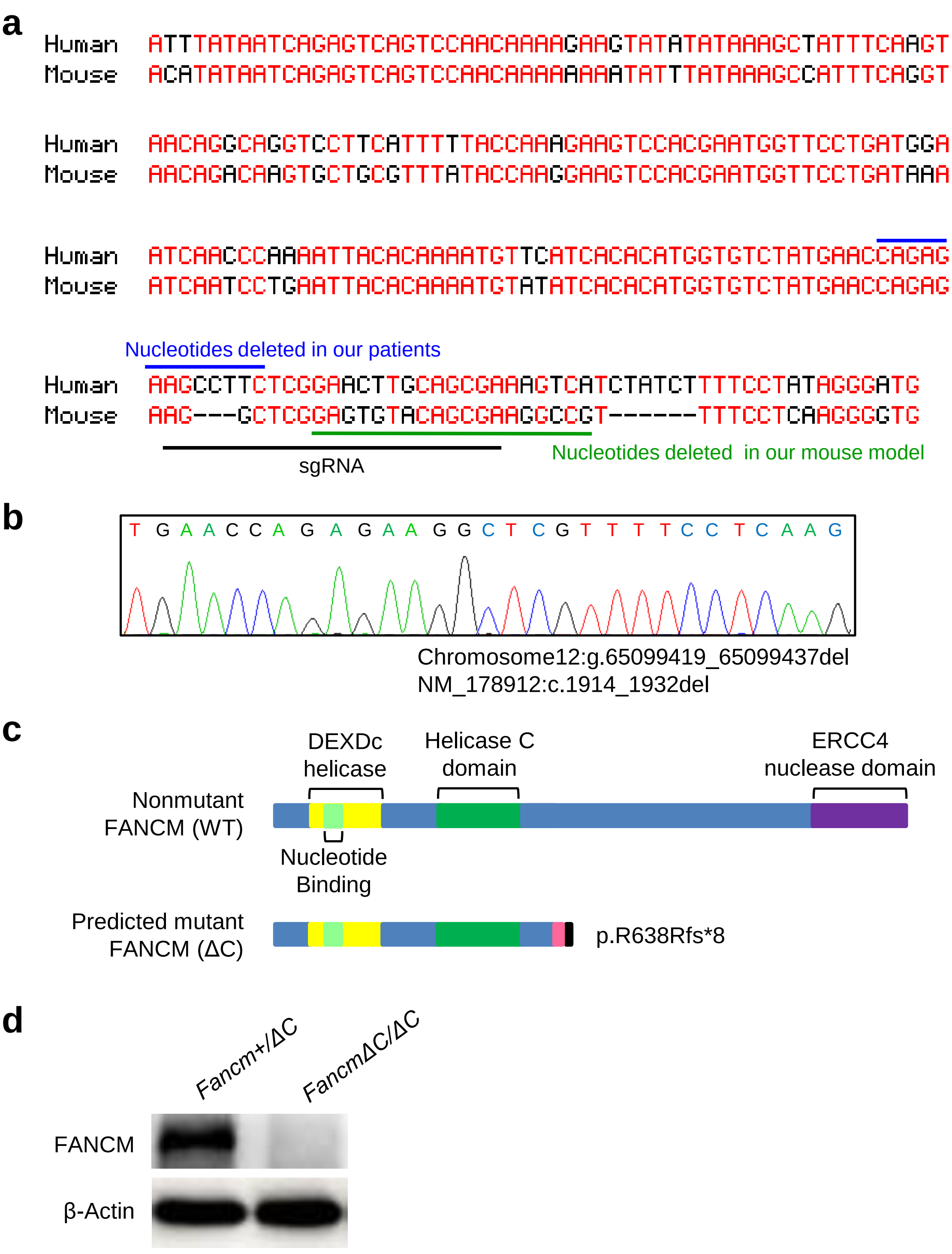

Supplementary Figure S5. Generation of the *Fancm* mutant mouse model carrying a homozygous mutation p.Arg638Argfs*8 equivalent to the *FANCM* mutation in our patients. (**a**) Sequence alignment of exon 11 of human *FANCM* and mouse *Fancm*. Conserved nucleotides are marked in red. The positions of the sgRNA for CRISPR/Cas9-mediated genome editing are underlined in black. The nucleotides deleted in our patients are below the blue line and the nucleotides deleted in mouse model are underlined in green. (**b**) Sequence chromatograms of the homozygous c.1914_1932del mutation in *Fancm*$^{\Delta C/\Delta C}$ mice. (**c**) The wild-type mouse FANCM protein (WT) and the predicted mutant mouse FANCM protein lacking the C-terminus (ΔC). The c.1914-1932del mutation causes a frame shift, resulting in a predicted truncated protein of 645 aa (p. R638Rfs*8). DEXDc：DEAD-like helicase domain. (**d**) Western blot in *Fancm*$^{+/\Delta C}$ mice and and *Fancm*$^{\Delta C/\Delta C}$ mice using an antibody recognizing the FANCM c-terminus. β-Actin served as a loading control.

Supplementary Figure S6

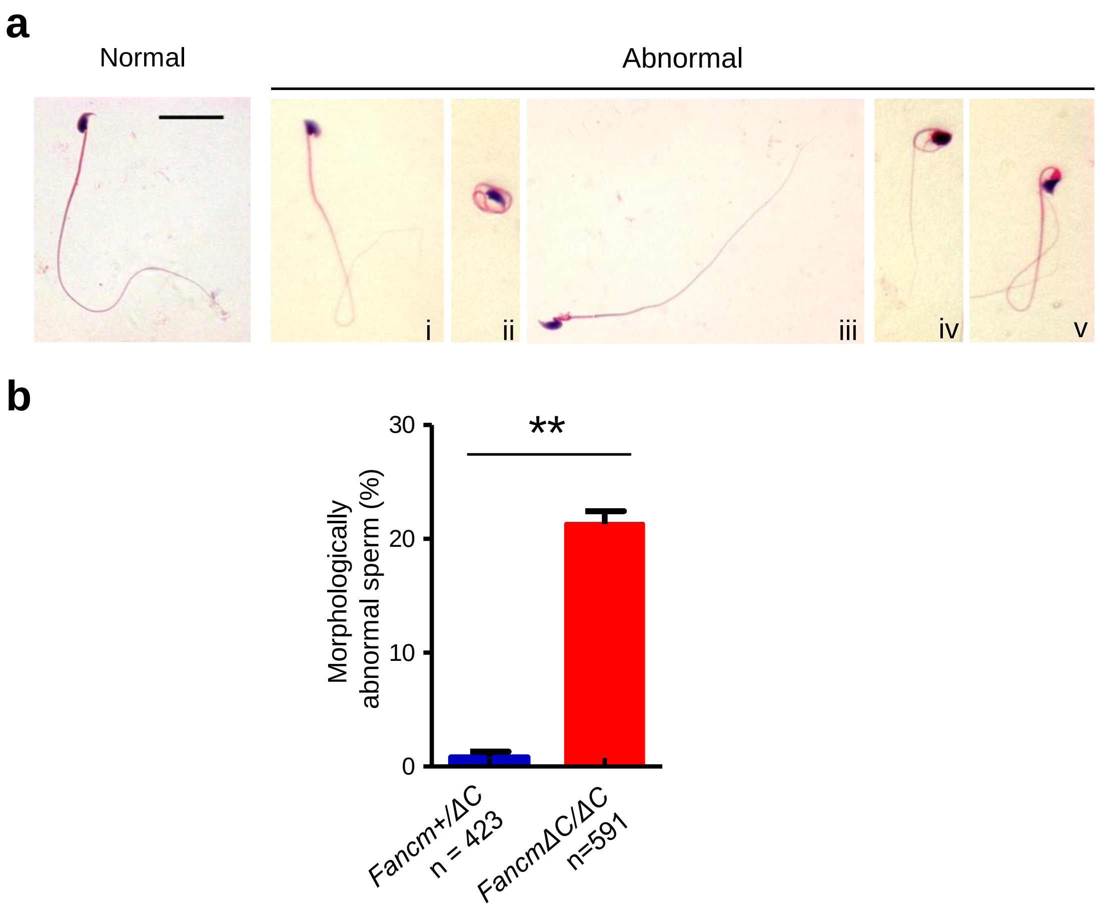

Supplementary Figure S6. Increased morphologically abnormal sperm in *Fancm*$^{ΔC/ΔC}$ mice. (**a**) Representative images of sperm with abnormalities in the head (i), tail (ii), neck (iii), head & tail (iv), or head & neck (v) in *Fancm*$^{ΔC/ΔC}$ mice. Sperm smears were prepared from epididymides and were stained with H&E. Scale bar, 20 μm. (**b**) Quantification of the abnormal sperm in *Fancm*$^{+/ΔC}$ mice and *Fancm*$^{ΔC/ΔC}$ mice. Data are represented as mean ± SEM from at least two experiments; n, the number of sperm scored. $^{**}p < 0.01$; Student's *t*-test.

Supplementary Figure S7

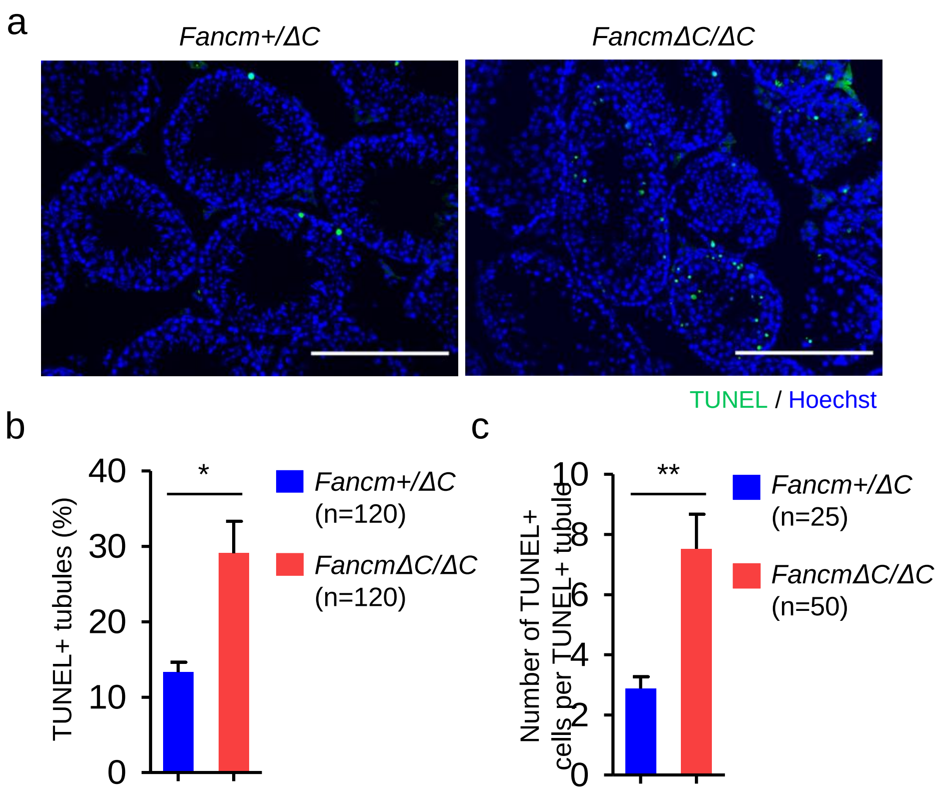

Supplementary Figure S7. Increased apoptosis in *Fancm*$^{\Delta C/\Delta C}$ testes. (**a**) Representative testicular sections stained for TUNEL. (**b**) and (**c**) Frequencies of TUNEL^{+} tubules (**b**) and average numbers of TUNEL^{+} cells per TUNEL^{+} tubule (**c**) in *Fancm*$^{+/\Delta C}$ mice and *Fancm*$^{\Delta C/\Delta C}$ mice. Data are represented as mean ± SEM from at least three mice; n, the number of tubules scored. *$p < 0.05$; **$p < 0.01$; Student's *t*-test. Scale bar, 200 μm.

Supplementary Figure S8

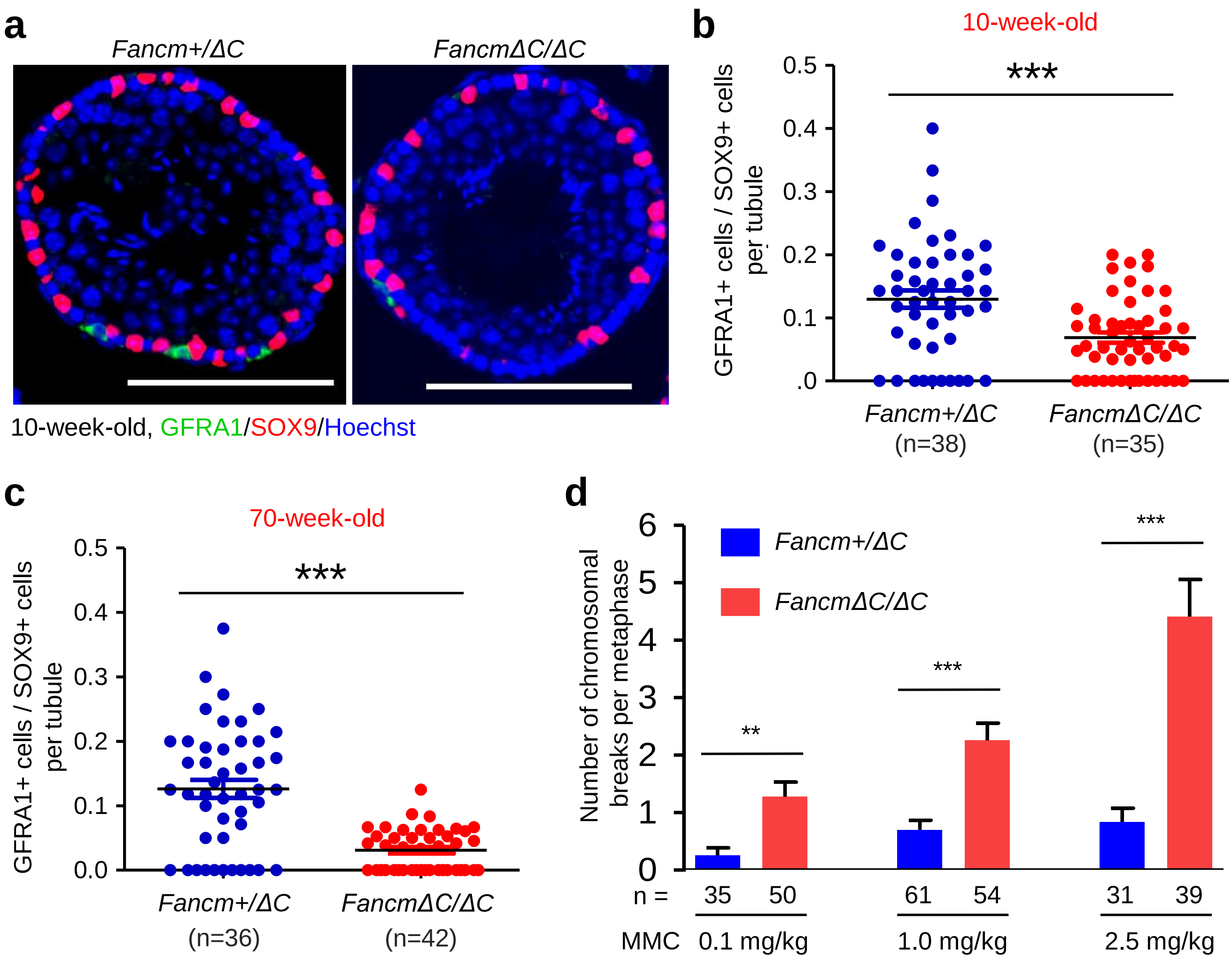

Supplementary Figure S8. Spermatogonial stem cells (SSCs) in adult *Fancm*$^{\Delta C/\Delta C}$ mice undergo a progressive loss with age and show hypersensitive to interstrand crosslinks. (**a**) Representative seminiferous tubules from 10-week-old *Fancm*$^{+/\Delta C}$ mice and *Fancm*$^{\Delta C/\Delta C}$ mice stained for GFRA1 (a marker of SSCs) and SOX9 (a Sertoli cell marker). Scale bar, 100 μm. (**b**) The ratios of SSC number to Sertoli cell number per seminiferous tubules in 10-week-old mice. Each dot represents the ratio of SSC number to Sertoli cell number in one seminiferous tubule. n, the number of tubules scored. (**c**) Reduced number of SSCs in tubules of *Fancm*$^{\Delta C/\Delta C}$ mice when compared to those of *Fancm*$^{+/\Delta C}$ mice at 70 weeks old. n, the number of tubules scored. (**d**) Deficient repair of MMC-induced chromosomal damages in spermatogonial metaphases of 10-week-old *Fancm*$^{\Delta C/\Delta C}$ mice. n, the number of cells scored. Data are represented as mean ± SEM from at least two experiments. **$p < 0.01$; ***$p < 0.001$; Student's *t*-test.

Supplementary Figure S9

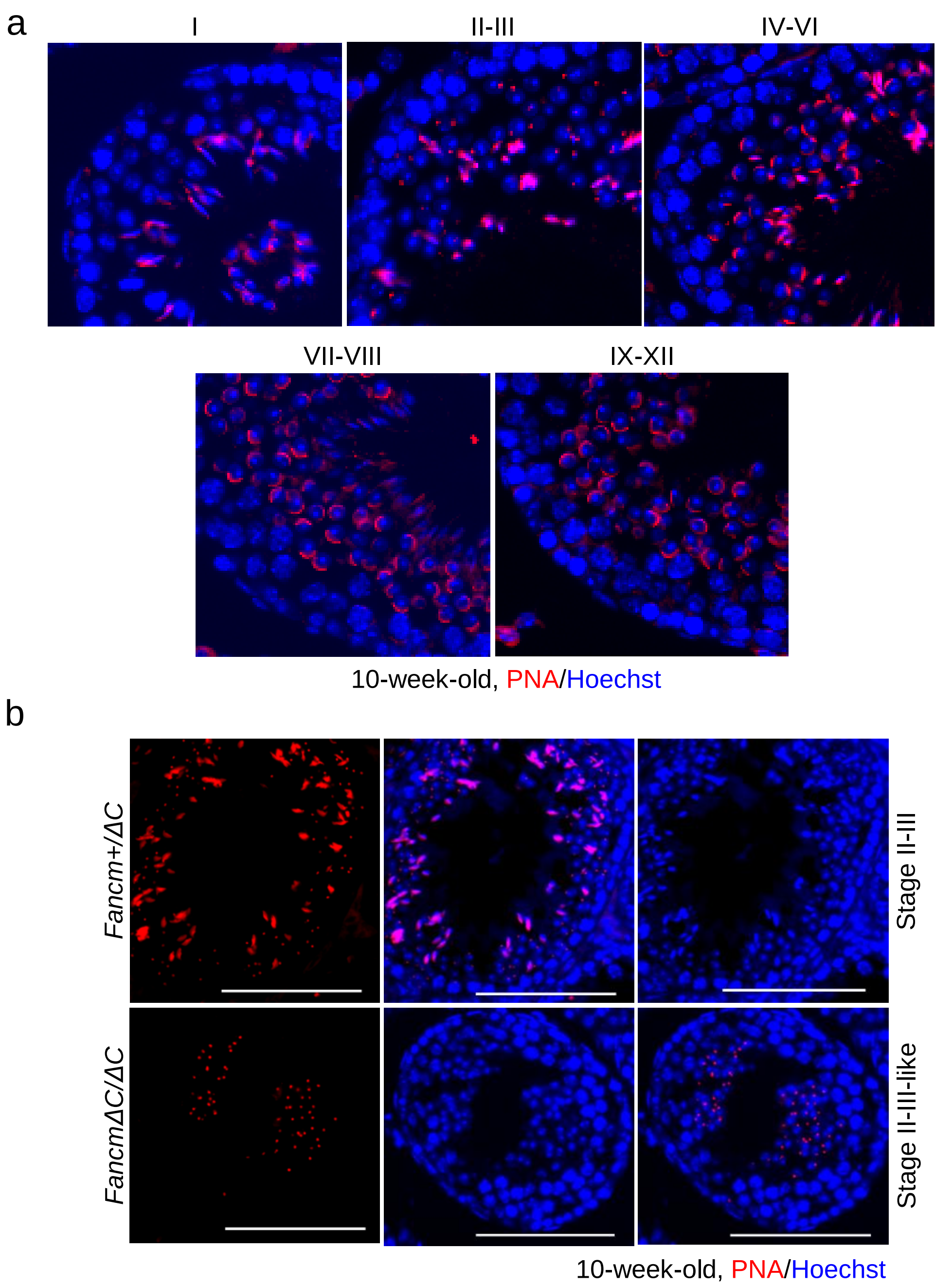

Supplementary Figure S9. Seminiferous tubule staging based on PNA staining pattern of mouse testicular sections. (**a**) Representative images of the five groups of spermatogenic stages in *Fancm*$^{+/\Delta C}$ mice. The spermatogenic stages were divided into five groups based on the type of spermatids and the shape of acrosomes in anti-PNA-stained testicular sections: I, II-III, IV–VI, VII-VIII and IX–XII. (**b**) Representative images of stage II-VI-like seminiferous tubules stained for PNA in *Fancm*$^{+/\Delta C}$ and *Fancm*$^{\Delta C/\Delta C}$ mice. A typical stage II-III tubule from *Fancm*$^{+/\Delta C}$ mice was shown, which contains both round and elongated spermatids, but the tubule at the same stage from *Fancm*$^{\Delta C/\Delta C}$ mice contains only round spermatids, which has not been observed in control mice and was named as stage II-III-like.

2. Supplementary Tables

Supplementary Table S1. List of software used for predicting the deleteriousness of mutations.

Number	Software name	PMID
1	SIFT	22689647
2	PolyPhen 2 HDIV	20354512
3	PolyPhen 2 Hvar	20354512
4	LRT	19602639
5	MutationTaster	24681721
6	MutationAssessor	21727090
7	FATHMM	23033316
8	MetaSVM	25552646
9	MetaLR	25552646
10	GERP++	21152010
11	SiPhy	21993624
12	PROVEAN	23056405
13	fathmm-MKL	25583119
14	DDIG	25573915; 23497682
15	VEP	27987178

Supplementary Table S2. Mutations identified by WES in patient IV:1 and his father III:1 were detected by Sanger sequencing in all the available family members.

Gene	Mutation		III:1	III:2	IV:1	IV:2	IV:3	IV:4
	Predicted at cDNA level	Predicted at protein level						
DSG1	c. 2515G>A	p.E839K	+/M	+/M	M/M	+/M	M/M	M/M
EIF3G	c.840+4_840+ 7GGCAdel	Not Determined	+/M	+/M	M/M	+/M	+/M	+/+
FANCM	c.1946_1958del	p. P648Lfs16*	+/M	+/M	M/M	M/M	M/M	+/M
IMMT	c. 878C>T	p.A293V	+/M	+/+	+/M	+/+	+/M	+/M
TAF7L	c.1047_1052delGG ATGA	p.353_354 delED	+/M	+/Y	M/Y	M/Y	M/Y	M/Y
STT3B	c.1677C>G	p.S559R	+/M	+/M	M/M	+/M	M/M	M/M
ZNF496	c.157G>T	p.A53S	+/M	M/M	M/M	M/M	+/M	ND

cDNA, complementary DNA; +, wild-type allele; M, mutant allele; ND, undetermined.

Table S3. Antibodies used in this study.

Protein	Company	Catalog number	Dilution
GFP	Abmart	M20004	WB：1:3000
FANCM	Novus Biologicals	NB110-98674	WB: 1:1000
	Kind gift from Fanconi Anemia Research Fund	FANCM-2	WB: 1:1000
FANCD2	Novus Biologicals	NB100-182	WB: 1:10000<br>IF: 1:500
γH2AX	Millipore	05-636	WB: 1:2000<br>IF: 1:500
PNA	Molecular probes	L32458	IF: 1:100
ACTB	Abcam	ab8227	WB: 1:10000
GFRA1	R&D	AF560	IF: 1:50
SOX9	Millipore	AB5535	IF: 1:400

Supplementary Table S4. Fertility test of *Fancm*$^{+/+}$, *Fancm*$^{+/\Delta C}$ and *Fancm*$^{\Delta C/\Delta C}$ mice.

	No. of male mice tested	No. of fertile males (%)	Litters	Pups per litter
***Fancm*$^{+/+}$**	4	4 (100)	27	7.56 ± 0.25*
***Fancm*$^{+/\Delta C}$**	7	7 (100)	47	7.36 ± 0.24*
***Fancm*$^{\Delta C/\Delta C}$**	7	6 (85.71)	29	2.90 ± 0.22

8 to 12-weeks old *Fancm*$^{+/+}$, *Fancm*$^{+/\Delta C}$, and *Fancm*$^{\Delta C/\Delta C}$ male mice were each housed with an 8-week old wild-type C57BL/6 female mice for 6 months. The litter numbers and pups per litter were recorded. Student's *t*-tests were performed between the numbers of pups per litter of *Fancm*$^{\Delta C/\Delta C}$ mice and those of *Fancm*$^{+/+}$ or *Fancm*$^{+/\Delta C}$ mice. Data are expressed as mean ± SEM. $*p < 0.001$.

Supplementary Table S5. Blood test results of *Fancm*$^{\Delta C/\Delta C}$ mice show no significant difference from those of 10-week-old *Fancm*$^{+/+}$ and *Fancm*$^{+/\Delta C}$ mice.

	***Fancm*$^{+/+}$**	***Fancm*$^{+/\Delta C}$**	***Fancm*$^{\Delta C/\Delta C}$**	*p* value[a]	*p* value[b]
**Hemoglobin (g/dl)**	12.84 ± 0.30	12.10 ± 0.13	12.00 ± 0.18	0.0585	0.6652
**RBC count (x10^{12}/L)**	8.95 ± 0.20	8.73 ± 0.12	8.57 ± 0.15	0.1999	0.4488
**HCT (%)**	41.28 ± 0.81	39.33 ± 0.25	39.58 ± 0.59	0.1497	0.7101
**MCV (fl)**	46.18 ± 0.90	45.08 ± 0.47	46.18 ± 0.57	0.9966	0.1855
**MCH (pg)**	14.34 ± 0.16	13.88 ± 0.14	14.00 ± 0.08	0.1249	0.4646
**MCHC (g/dl)**	31.10 ± 0.38	30.78 ± 0.32	30.30 ± 0.25	0.1506	0.3120
**WBC (x10^{9}/L)**	11.06 ± 1.34	11.85 ± 0.68	11.67 ± 0.41	0.7046	0.8350
**Neutrophils (%)**	16.94 ± 2.92	21.55 ± 3.22	16.60 ± 0.39	0.9214	0.1775
**Lymphocytes (%)**	62.76 ± 2.98	55.38 ± 4.51	59.28 ± 1.94	0.3837	0.4569
**Eosinophils (%)**	3.04 ± 0.34	3.48 ± 0.63	3.15 ± 0.19	0.7994	0.6415
**Monocytes (%)**	17.22 ± 0.91	19.6 ± 1.31	20.93 ± 2.09	0.1216	0.6097
**Platelets (X10^{9}/L)**	1446 ± 119.56	1445 ± 137.78	1454 ± 230.85	0.9747	0.9730

Student's-*t* tests were performed between 10-week-old *Fancm*$^{\Delta C/\Delta C}$ and *Fancm*$^{+/+}$ mice[a] or between *Fancm*$^{\Delta C/\Delta C}$ and *Fancm*$^{+/\Delta C}$ mice[b]. At least four mice were examined per group. Data are expressed as mean ± SEM.

Supplementary Table S6. Primer information used in this study.

**Primer information for amplification and Sanger sequencing of the mutations identified by WES**	
FANCM mut- F	CAGAGTCAGTCCAACAAAAG
FANCM mut-R	TACCAGTGTCTCTCCATTTG
DSG1 mut-F	GGAGAAGACCTGGATGACAG
DSG1 mut-R	AGAGCTTGGTGCTATTACCC
EIF3G mut-F	CCGTGTCACCAACTTGTCAG
EIF3G mut-R	GAGGATGAGGTGGTCGTAGC
ZNF496 mut-F	ATCCCATCATGCCCACAGCC
ZNF496 mut-R	TCCCGTTCCAGTGCCTCCAC
IMMT mut-F	TTCAATCTTGTTGGCCTATC
IMMT mut-R	TCCTAATGCCAATTCAGAAC
STT3B mut-F	CTTTAGGCAGGTAAAGTGAG
STT3B mut-R	CACGTGATTATTACACATGC
TAF7L mut-F	AGTATGAGTCATTTGCAGAG
TAF7L mut-R	GCTTTGAGTCTAATGTATGC
**Primer information for constructing FANCM_CDS-EGFP and FANCM-MUT_CDS-EGFP vectors**	
FANCM_GFP_F	GAACCGTCAGATCCGCTAGCATGAGCGGACGGCAAAGAACGC
FANCM_GFP_R	TCCTCGCCCTTGCTCACCATTATATCAGATTTCAGTCTATC
FANCM-mu-F	GAAGGCTTCTCTGGTTCATAGACACCATGTGTGATG
FANCM -mu-R	GTCTATGAACCAGAGAAGCCTTCTCGGAACTTGCAGCGAAAGTC
pEGFP-N1-F	ATGGTGAGCAAGGGCGAGGA

p-N1-R	GCTAGCGGATCTGACGGTTC
**Primer information for constructing EGFP-FANCM_CDS and EGFP-FANCM-MUT_CDS vectors**	
GFP-FANCM-F	GCATGGACGAGCTGTACAAGATGAGCGGACGGCAAAGAACGC
GFP-FANCM-R	GATCTAGAGTCGCGGCCGCTTTATATATCAGATTTCAGTC
FANCM-mu-F	GAAGGCTTCTCTGGTTCATAGACACCATGTGTGATG
FANCM -mu-R	GTCTATGAACCAGAGAAGCCTTCTCGGAACTTGCAGCGAAAGTC
p-N1-F	AGCGGCCGCGACTCTAGATC
P-GFP-C-R	CTTGTACAGCTCGTCCATGC
**Primer information for producing *Fancm*$^{-/-}$ HEK293T cells**	
Fancm-exon2-KO-sg1-U6-A	CCGGCGGAAACTATGCTTATTGCC
Fancm-exon2-KO-sg1-U6-S	AAACGGCAATAAGCATAGTTTCCG
Fancm-exon2-KO-sg2-U6-A	CCGGTCTACACAAGCTTCCACC
Fancm-exon2-KO-sg2-U6-S	AAACGGTGGAAGCTTGTGTAGA
Fancm-exon2-KO-Check-F	CCTAGATAAGTGCCAGCTTTG
Fancm-exon2-KO-Check-R	GACACTAATTATCTGGTTAG
**Primer information for generating *Fancm*$^{\Delta C/\Delta C}$ mice**	
mFancm-1946-1958-sgRNA-T7-A	TAGGAGGCTCGGAGTGTACAGCGA
mFancm-1946-1958-sgRNA-T7-S	AAACTCGCTGTACACTCCGAGCCT
mFancm-1946-1958-Check-F	GTTCTCCAGTACTTAAGTGA
mFancm-1946-1958-Check-R	GCTCCTGAGTTTATCTGAGG

3. Supplementary Methods

FANCM$^{-/-}$ cell generation and transfection

HEK293T cells were purchased from ATCC and maintained in DMEM medium (GIBCO) with 10% FBS (Hyclone). To generate *FANCM*$^{-/-}$ cells, two single guide RNAs (sgRNAs) targeting *FANCM* exon 2 were designed (sgRNA sequences are described in Supplementary Table S6) and cloned into pGL3-U6-sgRNA-PGK-puromycin (gift from Prof. X. Huang).[1] The sgRNA plasmids were co-transfected into HEK293T cells with pST1374-NLS-flag-linker-Cas9. Twenty-four hours later, cells were seeded into p60 dishes and cultured for 15 days in medium with blasticidin (8 μg/ml) and puromycin (2 μg/ml). Single colonies were picked, cultured in new dishes and then screened by Sanger sequencing for clones with *FANCM* null mutations (*FANCM*$^{-/-}$). The identified *FANCM*$^{-/-}$ clones were used for further experiments.

The human *FANCM* cDNA was a gift from Prof. M Teng.[2] Mutations were introduced by overlap extension PCR, and the coding sequences of wild-type or mutant *FANCM* were cloned into p*EGFP*-N1 vector. Primer sequences are listed in Supplementary Table S6.

Transfections were performed using Lipofectamine® 3000 Reagent (Thermo Fisher Scientific) according to the manufacturer's instructions, 12 hours after plating of cells.

Immunostaining and Western blot of cultured cells

Cells were grown on coverslips and treated with MMC (50 ng/ml for 12 hrs for γH2AX detection and 200 ng/ml for 24 hrs for FANCD2 detection). Immunostaining

with the antibodies described in Supplementary Table S3 was performed as we previously described.[3] Antibodies used in this study are listed in. For Western blot, HEK293T cells were treated with MMC as described above and cells were then lyzed with SDS lysis buffer and boiled for 10 min. The proteins were separated on a 10% SDS polyacrylamide gel by electrophoresis for Western blotting as we described.[3] Antibodies used are listed in Supplementary Table S3.

Chromosomal breakage assay in HEK293T cells

For MMC-induced chromosomal breakages analysis, HEK293T cells were cultured in DMEM supplemented with 10% FBS and 1% penicillin-streptomycin (Gibco), and treated with MMC (20 ng/ml) for 24 hr. Metaphase spreads were prepared using standard cytogenetic techniques described previously.[4] Chromosomal breaks were quantified by scoring at least 35 metaphases per culture.

Generation of *Fancm*$^{\Delta C/\Delta C}$ mice

Fancm$^{\Delta C/\Delta C}$ mice with a truncated FANCM protein analogous to that in our patients were generated by one-cell embryo injection using CRISPR/Cas9 genome editing tools.[1,5] A single guide RNA (sgRNA) targeting *Fancm* DNA sequences that were homologous to the human DNA sequence around the mutation site in our patients was designed. sgRNAs were transcribed *in vitro* and co-injected with Cas9 mRNAs into B6D2F1 (C57BL/6×DBA/2J) zygotes.[1] DNA from target region of founders was amplified and the mutations were verified by Sanger sequencing. The female founder carrying a homozygous 19-bp-deletion mutation in *Fancm* was bred to a C57BL/6 male to produce *Fancm*$^{+/\Delta C}$ mice. Homozygotes (*Fancm*$^{\Delta C/\Delta C}$) were obtained by intercrossing

Fancm$^{+/ΔC}$ mice (from 3rd backcross). All the mice were maintained under specific-pathogen-free conditions in laboratory animal centre of University of Science and Technology of China (USTC).

Haematoxylin and eosin (H&E) staining and immunofluorescence staining

10-week-old and 70-week-old (for immunofluorescence staining by anti-GFRA1 antibodies) *Fancm*$^{+/ΔC}$ and *Fancm*$^{ΔC/ΔC}$ male mice were euthanized by cervical dislocation. Epididymides and testes were collected and fixed in 4% paraformaldehyde in PBS for immunostaining, or in Bouin's solution for H&E staining. The tissues were embedded into paraffin blocks for sectioning. H&E staining and immunostaining were performed on testicular sections as we described.[5,6] Vendors and catalog numbers of the antibodies are listed in Supplementary Table S3. The stages of tubules were determined based on the composition of germ cells and the morphology of germ cell nuclei, following the published staging scheme.[7] The groups of tubule stages were classified based on the shape of acrosomes in spermatids after PNA staining, following the published criteria.[8]

TUNEL staining

Fancm$^{+/ΔC}$ and *Fancm*$^{ΔC/ΔC}$ mice were euthanized by cervical dislocation. Testes were collected and fixed in 4% paraformaldehyde in PBS. The testicular tissue was then embedded into paraffin blocks for sectioning. TUNEL staining was performed on testicular sections according to instructions provided with the cell death detection kit (Roche) as we described.[9]

Sperm analysis

One cauda epididymis was removed from each 10-week-old mouse, incised with scissors, and incubated in 1 ml buffer containing 75 mM NaCl, 24 mM EDTA and 0.4% bovine serum albumin (Sigma) at 37°C for 1 hr to allow sperm release. Sperm were then counted with a hemocytometer. Sperm motility was analyzed as previously described.[10] Briefly, extracted sperm were centrifuged and incubated in HTF medium (Millipore) supplemented with 10% FBS (Hyclone) at 37°C for 5 min. Sperm samples were then diluted and analyzed using Hamilton Thorne's Ceros II system for sperm motility.

Hematoxylin & Eosin (H&E) staining of sperm

Sperm were extracted from epididymis and concentrated in FBS. Sperm smears were made onto microscope slides, air-dried, fixed in 4% paraformaldehyde in PBS, and exposed to 1% Triton X-100, followed by staining with hematoxylin and eosin. Images were captured using a microscope (Nikon Eclipse 80i) equipped with a digital camera (Nikon DS-Ri1). The numbers of sperm with abnormalities in head, neck, tail, head & neck, or head & tail were quantified by examining at least 250 sperm per mouse.

Chromosome breakage assay in spermatogonial metaphase cells

10-week-old male mice were intraperitoneally injected with MMC at 0.1, 1.0 or 2.5 mg/kg. Twenty-four hours later, they were intraperitoneally injected with 4 mg/kg of colchicine and sacrificed 5 hr later.[11] Then, the spermatogonial chromosome preparations were prepared as previously described.[12] Briefly, the testes were removed, peeled of the testicular capsule. Cell suspensions were

prepared from isolated seminiferous tubule fragments in 2.2% (w/v) trisodium citrate dihydrate (isotonic solution) and centrifuged for 10 min at 800 rpm, followed by treatment with 1% (w/v) trisodium citrate dihydrate (hypotonic solution) for 12 min at 37°C and fixation in Carnoy's solution (75% methanol, 25% acetic acid) at 4°C. After three washes in fixative, chromosome preparations were made by dropping the cell suspension onto cold slides. Slides were dried and stained with Giemsa. Chromosomal breaks in spermatogonial metaphases (cells with 40 pairs of chromosomes) were quantified by scoring at least 31 cells for each group.

Routine blood test in mice

Peripheral bloods were sampled from lateral tail veins of 10-week-old mice and collected in EDTA-2K anticoagulant tubes (Anhui Heer). Bloods were analyzed using XT-1800i Automated Hematology Analyzer (Sysmex, Japan). Parameters that were collected are hemoglobin, red blood cell count (RBC), hematocrit (HCT), mean corpuscular volume (MCV), mean corpuscular hemoglobin (MCH), mean corpuscular hemoglobin concentration (MCHC), white blood cell count (WBC) (and percentages of neutrophils, lymphocytes, eosinophils and monocytes) and platelet count. At least four mice were examined for each group.

4. References

1. Shen B, Zhang W, Zhang J, et al. Efficient genome modification by CRISPR-Cas9 nickase with minimal off-target effects. *Nat Methods.* 2014;11(4):399-402.

2. Tao Y, Jin C, Li X, et al. The structure of the FANCM–MHF complex reveals physical features for functional assembly. *Nat Commun.* 2012;3:782.

3. Wang Z, Yin H, Zhang Y, et al. miR-214-mediated downregulation of RNF8 induces chromosomal instability in ovarian cancer cells. *Cell Cycle.* 2014;13(22):3519-3528.

4. Gupta A, Hunt CR, Hegde ML, et al. MOF phosphorylation by ATM regulates 53BP1-mediated double-strand break repair pathway choice. *Cell reports.* 2014;8(1):177-189.

5. Jiang X, Ma T, Zhang Y, et al. Specific Deletion of Cdh2 in Sertoli Cells Leads to Altered Meiotic Progression and Subfertility of Mice. *Biol Reprod.* 2015;92(3):79-79.

6. Ma H, Ng HM, Teh X, et al. Zfp322a Regulates mouse ES cell pluripotency and enhances reprogramming efficiency. *PLoS Genet.* 2014;10(2):e1004038.

7. Ahmed EA and de Rooij DG. Staging of mouse seminiferous tubule cross-sections. In: Keeney, S., ed. Cytological Methods. New York: Humana Press Publishing; 2009: Chapter 16.

8. Meistrich ML and Hess RA. Assessment of spermatogenesis through staging of seminiferous tubules. In: Carrell DT, and Aston KI, ed. Methods in Molecular Biology. New York: Springer Science+Business Media LLC Publishing; 2013: Chapter 27.

9. Jiang X, Ma T, Zhang Y, et al. Specific Deletion of Cdh2 in Sertoli Cells Leads to Altered Meiotic Progression and Subfertility of Mice. *Biol Reprod.* 2015;92(3):79-79.

10. Julio M. Castaneda, Rong Hua, Haruhiko Miyata, et al. TCTE1 is a conserved component of the dynein regulatory complex and is required for motility and metabolism in mouse spermatozoa. *Proc Natl Acad Sci.* 2017(114):27.

11. Adler I-D. Comparative cytogenetic study after treatment of mouse spermatogonia with mitomycin C. *Mutat Res Fundam Mol Mech Mutagen.* 1974;23(3):369-379.

12. Jiang L, Li T, Zhang X, et al. RPL10L Is Required for Male Meiotic Division by Compensating for RPL10 during Meiotic Sex Chromosome Inactivation in Mice. *Curr Biol.* 2017;27(10):1498-1505.
